# Digital Intervention Services to Promote HIV Self-Testing and Linkage to Care Services: A Bibliometric and Content Analysis—Global Trends and Future Directions

**DOI:** 10.3389/phrs.2024.1606354

**Published:** 2024-02-16

**Authors:** Frank Mhando, Marwa Nyankomo, Christa Hall, Kelia Olughu, Mbuzeleni Hlongwa, Samuel Janson, Love O. Idahosa, Genae Hatcher, Donaldson F. Conserve

**Affiliations:** ^1^ Johannesburg Business School, University of Johannesburg, Johannesburg, South Africa; ^2^ Ifakara Health Institute, Bagamoyo Research and Training Centre, Bagamoyo, Tanzania; ^3^ Milken Institute School of Public Health, The George Washington University, Washington, DC, United States; ^4^ School of Nursing and Public Health, University of KwaZulu-Natal, Durban, South Africa; ^5^ Public Health, Societies and Belonging, Human Sciences Research Council, Pretoria, South Africa; ^6^ Department of Economics, The University of Warwick, Coventry, United Kingdom

**Keywords:** digital health, mHealth, mobile health, linkage to care, HIV

## Abstract

**Objective:** The global burden of HIV remains a critical public health challenge, particularly in sub-Saharan Africa, home to over two-thirds of individuals living with HIV. HIV self-testing (HIVST) has emerged as a promising strategy endorsed by the World Health Organization to achieve UNAIDS targets. Despite its potential, challenges persist in linking self-testers to care post a positive result. Digital health interventions, including chatbots and mobile applications, offer innovative solutions to address this gap. However, a comprehensive bibliometric analysis of the collaboration and growth in the literature at the intersection of HIVST and digital interventions is lacking.

**Methods:** The study employs a bibliometric approach, leveraging data from the Web of Science, to analyze the characteristics, citation pattern and content of 289 articles spanning 1992–2023. The analysis involves performance assessment, scientific collaboration analysis, science mapping, and content analysis. Key bibliometric indicators, such as annual growth rate, citation impact, and authorship patterns, are explored. Collaboration patterns among countries, institutions, and authors are elucidated, and thematic mapping provides insight into the key research themes.

**Results:** The analysis reveals a dynamic and expanding field, with an annual scientific growth rate of 12.25%. Notable contributions come from diverse sources, including North America, Europe, and Africa. High-impact journals such as JMIR mHealth and uHealth play a crucial role in disseminating research findings. African authors, including Lebelonyane R, Ford N, and Lockman S, feature prominently, reflecting a positive trend in diverse authorship. Co-citation analysis highlights influential manuscripts, with systematic reviews dominating the top-cited articles. Collaboration analysis underscores strategic partnerships globally, particularly involving the United States, Australia, South Africa, and the United Kingdom.

**Conclusion:** This bibliometrics analysis provides a comprehensive overview of the digital health landscape in HIVST and linkage to care. It identifies key contributors, high-impact journals, and collaborative networks. The thematic map reveals nuanced research domains, including alcohol dependence, men’s health, outcomes, and user acceptance. The findings offer insights for researchers, policymakers, and practitioners, guiding future directions in the evolving intersection of HIVST and digital health interventions.

## Introduction

According to the World Health Organization (WHO), at the end of 2021 there were approximately 38.4 million HIV-positive individuals worldwide [1]. Moreover, the burden of the HIV epidemic continues to disproportionately affect sub-Saharan Africa wherein more than two-thirds of people living with HIV globally reside in the region [1]. HIV self-testing (HIVST) is recommended by the WHO as an innovative approach for attaining the Joint United Nations Programme on HIV/AIDS (UNAIDS) objectives of eliminating HIV by 2030 [2]. HIVST has received a lot of attention in the HIV testing field in recent years as a last-mile solution for meeting the UNAIDS 95-95-95 goals by 2030 [3]. In the interest of both individual and societal health, identifying HIV-positive people who are unaware of their status and connecting them to care are critical steps on the continuum leading to viral suppression. People who are aware of their status are generally less likely to engage in risky sexual behaviors and successful treatment adherence improves individual health outcomes and diminishes the risk of infecting others [4]. HIVST allows people to test at home and receive a preliminary result in private. It is particularly useful in settings with limited medical resources and low testing uptake among key populations to help facilitate earlier diagnosis and prevent further transmission [5]. These key populations in which HIV incidence has become more concentrated include men who have sex with men (MSM), people who inject drugs, and sex workers [6]. Moreover, HIVST has been shown to be cost-effective and widely accepted among those who may not otherwise get tested [5]. However, follow-up services to connect self-testers to care and treatment in the event of a positive result are lacking. Here, we define linkage to care as having a confirmatory HIV test after an HIVST positive result and completing a medical visit at a Care and Treatment Center if confirmed to have an HIV positive diagnosis [7, 8].

Self-testers who receive positive HIV results may benefit from innovative strategies that use digital technologies to help them access counseling and treatment. Digital tools such as chatbots, blockchain technologies, website-based, social media, mobile HIVST applications (apps), Short Message/Messaging Service (SMS), and digital vending machines (digital VMs) are examples of digital interventions used to improve HIV testing [1, 4]. Personalized, theory-based health material can be delivered with high fidelity and acceptability using mobile technologies and social media as an effective strategy to reach, engage, and retain key populations in HIV prevention and care initiatives [9]. The ubiquity and variety of technology use among younger people also offers several channels for practitioners to connect people to digital health interventions that increase HIV testing and linkage to care. Likewise, the COVID-19 pandemic has created additional obstacles for facility-based and in-person health services, emphasizing the value of virtual channels for facilitating flexible access to healthcare and conserving health system resources [10].

In a systematic review of digital innovations for HIV and Sexual Transmitted Infections (STI) control interventions from 1996–2017, all digital innovations were highly accepted and feasible with combination innovations significantly impacting antiretroviral therapy (ART) adherence, clinic attendance, partner notifications, and self-care [11]. Likewise, investigators examining the utility of social media interventions aimed at promoting HIV testing found that these interventions could be used to create online interactive communities to encourage HIV testing and treatment adherence, offer HIV testing services, generate further intervention materials, and provide a channel for participants to request HIVST kits [12]. For example, a randomized controlled trial evaluating a digital intervention on ART among MSM living with HIV in China found that those in the intervention arm receiving ART medication information and reminders, peer education, and support had a higher proportion of participants achieving optimal adherence than in the control arm [13].

Similarly, the first pilot HIV testing combination intervention in Nigeria targeting young MSM including social media outreach and virtual peer navigation demonstrated success with identifying new HIV cases by increasing uptake of HIV testing [14]. Within the iCare Nigeria (Intensive Combination Approach to Roll Back the Epidemic in Nigerian Adolescents) intervention, creating closed groups on various social media platforms (i.e., Grindr, WhatsApp, Facebook) enabled peer navigators to engage young MSM with posts and discussions on health topics including sexual orientation and gender identity, correct condom use, pre exposure prophylaxis, and the importance of HIV testing and early ART. Additionally, investigators examining the effects of mobile health (online and smartphone apps) to encourage HIV-preventative behaviors among MSM found increased HIV testing and decreased condomless anal intercourse with the use of messaging, social media, and combined technology modalities [15]. HIVST innovations with digital support also offer a great opportunity to reduce stigma and confidentiality difficulties among hard-to-reach populations, and improve connectivity to care [7].

Considering the importance of HIV testing to prevent transmission and achieving UNAIDS goals, the literature concerning HIV testing and linkage to care has been increasing over the past several years. However, the trend of collaboration and growth of HIVST literature with digital intervention have not been explored in a bibliometric analysis. Previous bibliometric studies [11, 12, 16, 17] have explored the trend and the growth of the HIV/AIDS literature in general. Still, unfortunately, there is a scarcity of bibliometric studies investigating and reporting advances of HIVST with digital supports to enhance linkage to care. Reflecting the global scientific effort to address the epidemic, bibliometric studies of research output in international settings documenting the productivity and collaboration is needed. This paper aims to fill this gap with a quantitative bibliometric analysis on digital health in HIV Self-testing and linkage to care services. A quantitative review enhances the understanding of this research field and further informs the scientific debate on this topic. Specifically, using the main procedures of the bibliometric method (performance analysis, scientific collaboration analysis and science, mapping), the work aims to 1) quantify the research field and describe its main outputs and evolution; 2) analyze the collaboration practices and map the social structure of the field; 3) define the intellectual structure and understand the main conceptualizations and theoretical approaches; and 4) identify the most investigate themes and propose future avenues for research.

## Methods

Since the main aim of this study is to assess the current level of knowledge development on digital health services, HIVST and linkage to care, we opted for a bibliometric approach which can be defined as “the measurement of all aspects related to the public and reading of books and documents” [18]. Bibliometric analysis has been increasingly used in both social sciences [19], and with specific reference to the health field [20, 21]. It is based on the statistical measurement of science, scientists, or scientific activity and it is, therefore, considered a more objective and reproducible method with which to develop a review process compared to other techniques [22, 23]. The process of data collection and data analysis is detailed below.

### Data Collection

**FIGURE 1:**
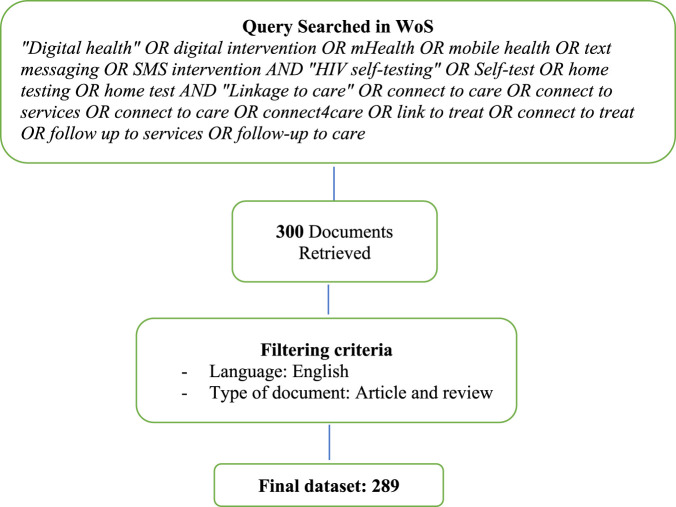
Data collection flowchart (Tanzania, 2023).

Data were retrieved from the Web of Science (WoS) and specifically from the Science Citation Index Expanded (SCI expanded) and the Social Science Citation Index (SSCI) [25, 26]. It is a world-leading publisher-independent global citation database with a powerful research engine that can track contents across “disciplines and time from over 1.7 billion cited references from over 159 million records.” It contains enough data to make it suitable for most bibliometric analysis and is already included in most university subscriptions, so it is immediately available to researchers working in academic settings [25]. The search criteria were (*“Digital health” OR digital intervention OR mHealth OR mobile health OR text messaging OR SMS intervention*) *AND* (*“HIV self-testing” OR Self-test OR home testing OR home test*) *AND* (*“Linkage to care” OR connect to care OR connect to services OR connect to care OR connect4care OR link to treat OR connect to treat OR follow up to services OR follow-up to care*) (that is, all fields). The search includes correlated words for digital health, HIV self-testing and linkage to care. The query was launched on October 29, 2023 and resulted in the retrieval of 300 documents. The search was then refined by language (English) and document type (article and review) [26]. No limitation on time was selected in order to gather the evolution over time since its first appearance in literature. The filtering stage produced 289 documents. To avoid including papers that were not related to the topic, i.e., not containing the concept of digital health, HIV self-testing and linkage to care, the collection was screened in terms of titles and abstracts. This phase was carried out by the corresponding author and then checked by two other authors. This screening thus reduced the risk of including unrelated articles or, conversely, excluding pertinent ones. The final sample is made up of 289 documents as shown in Figure 1 below.

### Data Analysis

In order to gain insights into the structure, social networks, and pertinent topics of a scientific field, bibliometric techniques are concentrated on the examination of a document’s bibliographic qualities, commonly referred to as “metadata,” such as authors, citations, collaboration, and key phrases [20, 22, 24]. The Bibliometrix, an open-source application in R for bibliometric analysis was used. The sample documents retrieved from WoS in BibTeX format were loaded into R using Bibliometrix, and data were converted into an R dataframe within Biblliometrix for further analysis [22]. The first three stages of analysis—performance analysis, collaboration analysis, and science mapping—were made possible as a result followed by content analysis.

### Performance Analysis

A performance analysis highlights the sample characteristics and measures its main performances by quantifying the research field (the number of published documents, the number of received citations), identifying the most important (most cited, most productive, etc.) actors, and evaluating groups of scientific actors (countries, universities, departments, researchers) and the impact of their activity [25, 27, 28]. At this stage, a citation analysis was also performed. A citation analysis is based on the hypothesis that authors cite documents considered most important in the development of their research. Frequently cited studies are expected to have a greater influence on the research field than those less frequently cited [23, 29, 30]. To ensure the accuracy of the data, the references were checked to ensure that they were written in the same way in all the documents. Various metrics, including the number of documents, citations, and average citations per document were calculated to measure the field’s performance. The annual growth rate is calculate using compound annual growth rate (CAGR) formula;

$$CAGR={\left(\frac{\text{Ending}\;\text{Value}}{\text{Beginning}\;\text{Value}}\right)}^{\frac{1}{\text{Number}\;\text{of}\;\text{Years}}}-1$$

The average age of the document is calculated by taking the sum of the ages of all documents (time since publication) and dividing it by the total number of documents. The average citations per document is calculated by dividing the total number of citations across all documents by the total number of documents.

### Collaboration Analysis

A scientific collaboration analysis was then carried out in order to highlight the most relevant relations between the actors (i.e., authors and countries). Scientific collaboration analysis is widely used in different strands of research, e.g., [27, 30–33], in order to identify the social structure of the field. This is achieved via a social network analysis where the network’s nodes are the authors, their institution or country to which the institutions belong, and the edges (links) are established according to the nodes who co-authored an article. Nodes represented authors, institutions, or countries and edges represented co-authorship relationships. The resulting network was analyzed to identify collaboration patterns among authors and countries.

### Science Mapping

The science mapping was performed through a co-citation analysis and co-word analysis. Science mapping “is a spatial representation of how disciplines, fields, specialties, and individual papers or authors are related to one another” [34]. Science mapping examines the *relationships* between research constituents [26, 34, 35]. The analysis pertains to the intellectual interactions and structural connections among research constituents. The techniques for science mapping include citation analysis, co-citation analysis, bibliographic coupling, co-word analysis, and co-authorship analysis. Such techniques, when combined with network analysis, are instrumental in presenting the bibliometric structure and the intellectual structure of the research field [35]. The co-citation analysis was used to capture the intellectual structure of the field. Co-citation is defined as the frequency with which two documents are cited together in the literature and it assumes that documents are co-cited if they are conceptually close [23, 29, 36]. A co-word analysis is based on the idea that the co-occurrence of key terms (i.e., abstract, title or keywords) describes the content of the documents. This technique identifies and visualizes clusters that represent se-mantic or conceptual groups of different topics treated in the research field. Using the approach developed by Cobo et al. [34] the thematic clusters are visualized in a “strategic diagram” or map. Co-citation analysis captured the intellectual structure by analyzing how frequently two documents are cited together, revealing conceptual closeness. Co-word analysis identified thematic clusters by analyzing the co-occurrence of key terms (abstract, title, keywords) in documents. Strategic diagram visualized thematic clusters using a “strategic diagram” or map.

### Content Analysis

The content analysis was also conducted within the bibliometric analysis papers to provide a nuanced exploration of the landscape surrounding digital interventions in the context of HIV self-testing and linkage to care. By delving into the content of the document, the analysis unveils key patterns, challenges and strategies that shape the discourse in the dynamic filed. This analysis involved analyzing the documents qualitatively by delving into their content to unveil nuanced insights. Key patterns, challenges, and strategies discussed in the documents were identified and summarized.

## Results

### Performance Analysis: Sample Characteristics

**FIGURE 2:**
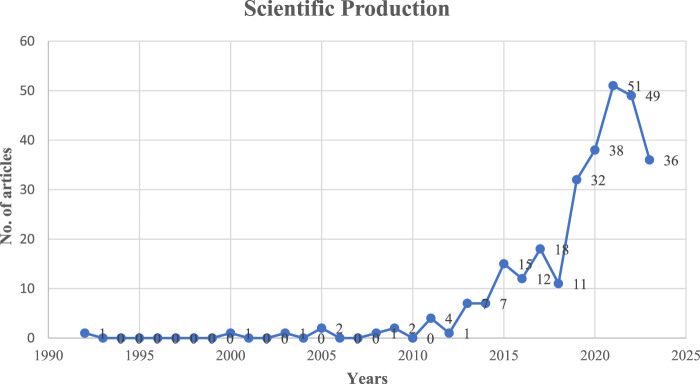
Annual scientific production (Tanzania, 2023).

The articles of the sample have been published from 1992 to 2023, comprising 289 documents from 149 sources, including journals and books (Table 1). This field is fairly new with an annual scientific growth rate of 12.25%, reflecting a dynamic and expanding dataset. On average, the documents are relatively recent, with an average age of 3.82 years. Each document receives an average of 16.57 citations, indicating a significant level of scholarly engagement. As shown in Figure 2, the first clear jump in the production of articles occurred in 2015 with 15 documents, then the biggest increase has occurred in 2021 with 51 articles.

**TABLE 1:** Main information about documents published on Digital intervention, HIV self-testing and linkage to care in the Web of Science (Tanzania, 2023).

Description	Results
Main Information About Data	
Timespan	1992–2023
Sources (Journals, Books, etc.)	149
Documents	289
Annual Growth Rate %	12.25
Document Average Age	3.82
Average citations per doc	16.57
References	1
Document Contents	
Keywords Plus	826
Author’s Keywords	952
Authors	
Authors	2,146
Authors of single-authored docs	1
Authors’ Collaboration	
Single-authored docs	1
Co-Authors per Doc	8.17
International co-authorships %	45.33
Document Types	
Article	264
Review	25

Authorship is very fragmented, with 2,146 authors, with single-authored documents accounting for 1, and a collaborative effort involving an average of 8.17 co-authors per document. Notably, 45.33% of these collaborations are international, highlighting a substantial level of global research collaboration.

The articles in the dataset were published in 149 journals, revealing a diverse distribution across various research domains, including medicine, management, business, and social sciences. Notably, only 24 journals 16.2% have published more than three articles, while 46 journals 31% have contributed more than two articles each, resulting in a mean of 1.9 article per journal. Strikingly, approximately 80% of the total documents are represented by a select journal, emphasizing their pivotal role in scholarly contributions. These top journals span diverse fields, with notable mentions being in health research, such as BMJ Open, and the public sector, exemplified by Public Management Review. This concentration of articles in specific journals underscores their significance as key players in disseminating research findings across different domains.

In examining the most cited manuscripts, several standout contributions emerge (Table 2). Topping the list is the work by Sharma et al. (2015) published in Nature [37], accruing a substantial 344 total citations with an impressive annual citation rate of 38.22. Following closely is Suthar et al. (2013) in PLOS Medicine [38], accumulating 280 total citations at a rate of 25.45 per year. Govindasamy et al. (2014) in the Journal of the International AIDS Society [39] is also noteworthy, with 188 total citations and an annual citation rate of 18.80. Interestingly, the top 10 manuscripts consist of five systematic reviews, four Trials and 1 empirical study. These manuscripts not only demonstrate high citation counts but also sustained impact over the years, as reflected in their normalized citation rates. Most of the top 10 articles were published in The Cochrane Database which primarily publishes reviews synthesizing evidence in healthcare. This analysis sheds light on the significant influence and enduring relevance of these top manuscripts in the scholarly landscape.

**TABLE 2:** Top manuscripts by citation (Tanzania, 2023).

Paper	DOI	Total citations (TC)	TC per year	Normalized TC
SHARMA M, 2015, NATURE	10.1038/nature16044	344	38.22	7.28
SUTHAR AB, 2013, PLOS MED	10.1371/journal.pmed.1001496	280	25.45	3.55
GOVINDASAMY D, 2014, J INT AIDS SOC	10.7448/IAS.17.1.19032	188	18.80	2.57
THOMSON H, 2013, COCHRANE DB SYST REV	10.1002/14651858.CD008657.pub2	168	15.27	2.13
BAKER DW, 2014, JAMA INTERN MED	10.1001/jamainternmed.2014.2352	143	14.30	1.96
MENDOZA JA, 2017, PEDIATR BLOOD CANCER	10.1002/pbc.26660	127	18.14	4.51
HE J, 2017, JAMA-J AM MED ASSOC	10.1001/jama.2017.11358	109	15.57	3.87
GOVINDASAMY D, 2015, J INT AIDS SOC	10.7448/IAS.18.1.20182	87	9.67	1.84
HIRSHBERG A, 2018, BMJ QUAL SAF	10.1136/bmjqs-2018-007837	80	13.33	3.35
LABHARDT ND, 2014, PLOS MED	10.1371/journal.pmed.1001768	71	7.10	0.97

The geographical distribution of papers suggests a diverse representation across various institutions and regions (Table 3). Institutions in North America, such as the University of Washington, the University of California, San Francisco, and the university of North Carolina, contribute significantly to the research output. In Europe, the London School of Hygiene and Tropical Medicine stands out as a prominent contributor. The University of Toronto represents a notable presence in Canada. From African continent, the University of Pretoria and Makerere University are noteworthy contributors, reflecting the research activities in South Africa and Uganda respectively. Monash University’s contribution highlights research from Australia. This international distribution underscores the collaborative and global nature of the research landscape, with institutions from different regions actively participating in advancing scientific knowledge.

**TABLE 3:** Top productive affiliations contributing to research articles (Tanzania, 2023).

Affiliation	Articles
University of Washington	33
London School of Hygiene and Tropical Medicine	23
University of California San Francisco	20
University of North Carolina	20
University of Toronto	17
University of Pretoria	15
Emory University	12
Duke University	11
Makerere University	11
Monash University	11

In regards with most productive journals Table 4 shows that JMIR MHEALTH AND UHEALTH emerges as a highly influential journal in mobile and ubiquitous health, boasting notable metrics with an h-index of 8, a g-index of 12, and an m-index of 1.143, since its inception in 2017. PLOS MEDICINE leads in total citations with 427, indicating significant recognition in the broader field of medicine. Both journals, focused on research protocols and clinical trials, respectively, exhibit notable impact metrics. This indicates the importance of journals specializing in methodological aspects and disseminating findings from clinical trials.

**TABLE 4:** Top productive journals contributing to the field of Digital health, HIV self-testing and linkage to care (Tanzania, 2023).

Element	h_index	g_index	m_index	TC	NP	PY_start
JMIR MHEALTH AND UHEALTH	8	12	1.143	182	12	2017
JMIR RESEARCH PROTOCOLS	7	12	0.778	150	14	2015
JOURNAL OF MEDICAL INTERNET RESEARCH	7	9	0.636	151	9	2013
PLOS MEDICINE	7	7	0.636	427	7	2013
TRIALS	7	9	1	97	11	2017
PLOS ONE	6	9	0.857	103	9	2017
BMC PUBLIC HEALTH	4	8	0.444	69	8	2015
JOURNAL OF THE INTERNATIONAL AIDS SOCIETY	4	5	0.4	300	5	2014
AIDS CARE-PSYCHOLOGICAL AND SOCIO-MEDICAL ASPECTS OF AIDS/HIV	3	3	0.333	59	3	2015
COCHRANE DATABASE OF SYSTEMATIC REVIEWS	3	3	0.273	244	3	2013

h_index -a measure of the journal’s scholarly impact based on the number of highly cited papers and the number of citations they received; g_index—a variant of the h_index that considers the distribution of citation across papers; m_index-a measure reflecting the ration of h-index to the number of years since the first publication; TC (total citation) -the total number of citations received by the journal; NP (Number of Publication) -the total number of publications produced by the journal; PY_start (Publication year start)-the year in which the journal publications started.

### Most Relevant Authors

Table 5 highlight the contribution of various authors to the body of work, with a notable focus on African authors. Lebolonyane R, Ford N, and Lockman S, both from Africa, have authored four article each, contributing to almost 30% to the total. The involvement of these African authors underscores their significant role in the research, contributing to substantial portion of the articles listed. This reflects positively on the representation of African researchers in the scholarly output, emphasizing their valuable contribution to the field. It’s encouraging to see diverse authorship and contribution in research, bringing perspectives from different regions to the forefront of academic endeavors.

**TABLE 5:** Most relevant authors contributing to the field of digital health, HIV self-testing and linkage to care (Tanzania, 2023).

Authors	Articles	Articles fractionalized
TANG WM	6	0.45
BACHANAS P	4	0.25
BUKUSI EA	4	0.43
FORD N	4	0.50
KLAUSNER JD	4	0.77
LEBELONYANE R	4	0.25
LOCKMAN S	4	0.25
SULLIVAN PS	4	0.61
TUCKER JD	4	0.34
AGOT K	3	0.35

### Collaboration Analysis

**FIGURE 3:**
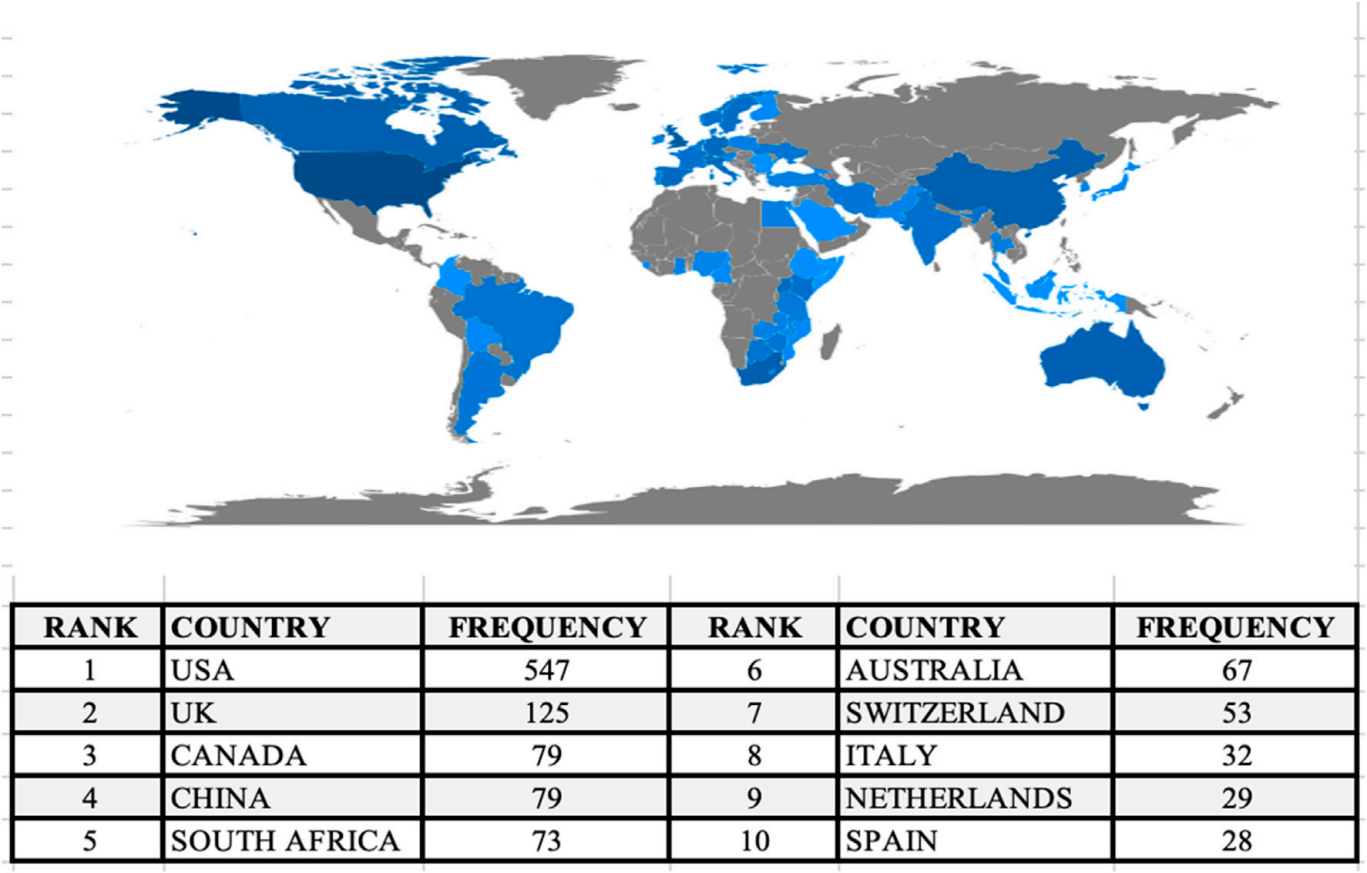
Scientific Production by Country. Map generated through “Biblioshiny” a shiny app providing a web-interface for Bibliometrix software (Tanzania, 2023).

With reference to various aggregation levels, this report provides a general overview of the scientific collaboration and research communities [37]. Authors and nations were used as the units of analysis in this study as shown in Figure 3.

### Cross-Country Collaboration

**FIGURE 4:**
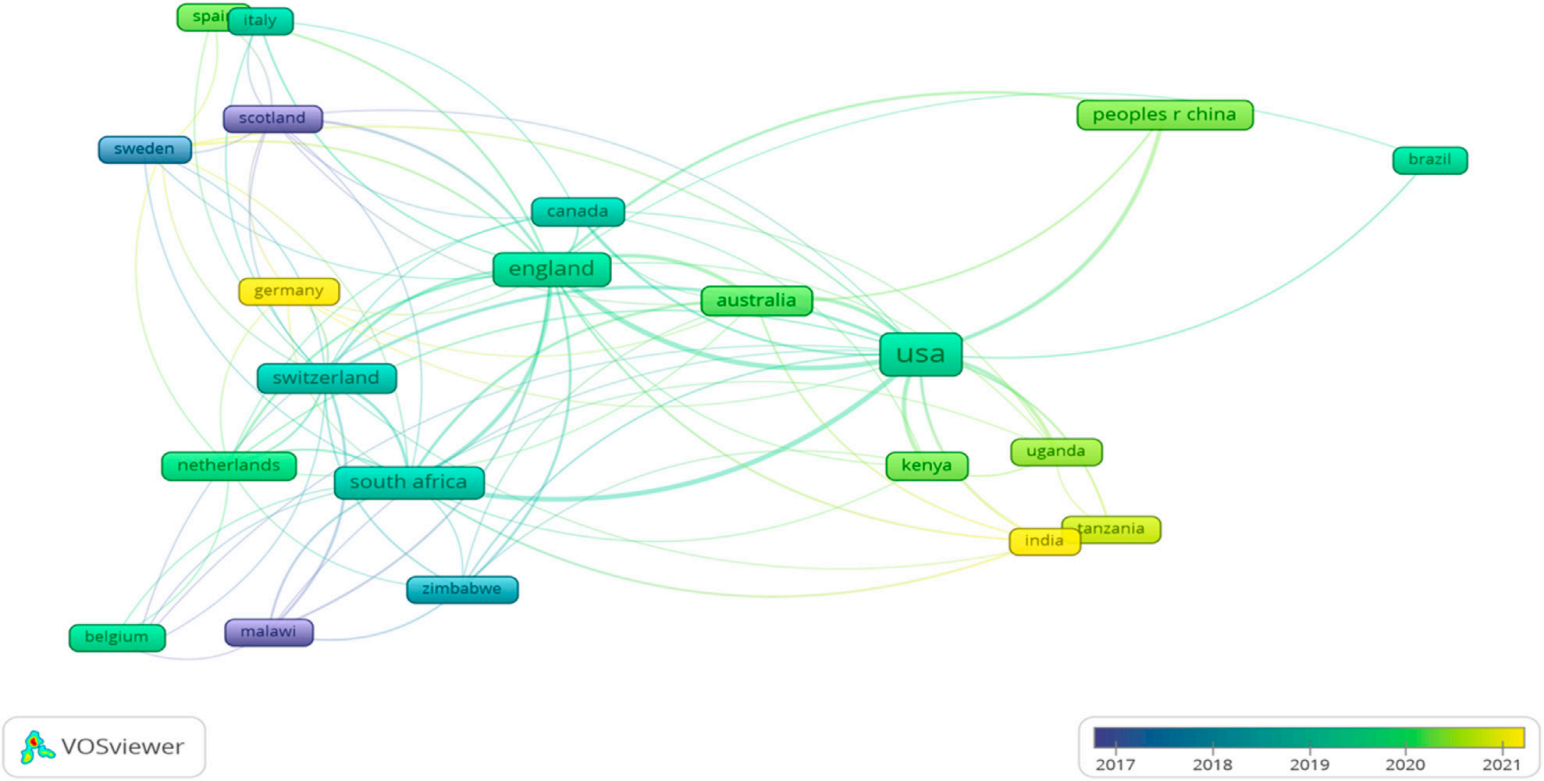
Countries’ Collaboration (Tanzania, 2023).

Table 6 and Figure 4 presents a comprehensive view of collaboration patterns among countries based on scholarly articles in the dataset. The United States emerges as a major contributor with 113 articles, of which 75 are single-country publications (SCP), and 38 involve collaborations with other nations (MCP). Notably, Australia engages in frequent collaborations, particularly with Argentina, India, and South Africa. Canada’s collaborations span diverse nations, including Italy, Switzerland, and Uganda. South Africa emerges as a key player with significant ties to Australia, Switzerland, and the United Kingdom. The United Kingdom, in turn, exhibits extensive collaborations with countries such as Australia, China, and South Africa. The United States participates in widespread collaborations, notably with Kenya, South Africa [41], and the United Kingdom (see Supplementary File S1). The collaborations suggest strategic partnerships and research network hubs, with certain countries like the United States, the United Kingdom, and Australia serving as key contributors to global research efforts. Collaboration within regions is also evident, such as the collaborations between countries in AFRICA (e.g., SOUTH AFRICA, NIGERIA, KENYA) and Europe (e.g., SWITZERLAND, GERMANY).

**TABLE 6:** Countries production and international collaboration patterns (Tanzania, 2023).

SN	Country	Articles	SCP	MCP	Freq	MCP_Ratio
1	United States	113	75	38	0.391	0.336
2	United Kingdom	26	9	17	0.09	0.654
3	China	19	9	10	0.066	0.526
4	South Africa	19	7	12	0.066	0.632
5	Canada	13	7	6	0.045	0.462
6	Switzerland	8	1	7	0.028	0.875
7	Netherlands	7	2	5	0.024	0.714
8	Australia	6	4	2	0.021	0.333
9	Brazil	5	2	3	0.017	0.6
10	Germany	5	2	3	0.017	0.6

Country, country of the corresponding author’s affiliation; Articles, number of articles per country of corresponding author’s affiliation; SCP, single country publication; MCP, multi country publication.

### Collaborations Among Authors

In order to understand any long-lasting collaborations among authors the co-authorship network (see Supplementary File S3) provide a detailed analysis. The node cluster analysis, based on betweenness, closeness, and PageRank centrality measures, reveals distinct patterns in the network. Cluster 1–6 authors such as “gloyd s,” “hughes jp,” “van heerden a,” and “van rooyen h" belong to clusters with low betweenness, indicating they might not serve as critical bridges between different parts of the network. High closeness within these clusters suggests strong internal connections among authors. Moderate PageRank values imply a moderate level of influence within their respective clusters. Clusters 7–9 authors in these clusters, such as “bukusi ea,” “agot k,” “camlin cs,” and “ayieko j,” exhibit moderate to high betweenness, suggesting potential bridging roles connecting different clusters or parts of the network. Moderate closeness and PageRank values indicate a balanced level of influence and connectivity. Cluster 10–11 authors like “tang wm,” “tucker jd,” “ezechi o,” and others in Cluster 10 have nodes with moderate to high betweenness, potentially serving as influential nodes connecting disparate parts of the network. In Cluster 11, authors with low betweenness but high PageRank, such as “bachanas p,” “lebelonyane r,” and “lockman s,” may hold significant influence within their respective clusters. The cluster analysis was conducted based on centrality measures such as betweenness, closeness and PageRank without specific details about the content of the nodes (areas of publication or their affiliation).

### Science Mapping

### Thematic Map: Co-word Analysis

A co-word analysis of author keywords helps to define a map of the main themes of the field. The centrality measures the degree of interaction of a network with other networks and is considered “as a measure of the importance of a theme in the development of the entire research field analyzed” [34]. The density measures the internal strength of the network and identifies the degree of development of a theme. The size of the cluster is given by the number of occurrences of the keywords that it contains and therefore by the number of linked papers (see Supplementary File S4).

In Cluster 1 (“alcohol”), terms such as “alcohol” and “dependence” suggest a focus on issues or studies related to alcohol dependence. This cluster has a moderate presence in the dataset, with a balanced level of centrality and density. Cluster 2 (“men”) encompasses a diverse set of terms related to men, HIV, and associated factors, reflecting a broad exploration of topics within this domain. The “Men” cluster is highly central and dense in the network, indicating its significance and frequent occurrence in the dataset. In Cluster 3 (“outcomes”), terms like “therapy” and “health” indicate a concentration on studies or discussions regarding health outcomes and therapeutic effectiveness. This cluster is moderately central with a higher density of connections, suggesting a focused theme with moderate frequency. Moving on to Cluster 4 (“care”), various terms related to healthcare, interventions, and care practices suggest a thematic focus on healthcare-related studies. The “Care” cluster is highly central and dense, indicating its prominence and frequent appearance in the dataset. Cluster 5 (“user acceptance”) incorporates terms related to user acceptance of health technologies and predictive models. This cluster has low centrality but very high density, suggesting it might be a niche topic with fewer occurrences. Cluster 6 (“behavior-change”) combines terms like anxiety, screening tools, and behavior change, hinting at studies or discussions on anxiety management and behavior change strategies. Finally, Cluster 8 (“nursing-home residents”) encompasses terms related to nursing-home residents, long-term care, and associated factors, pointing towards research in this specific healthcare context. Cluster 6, 7, and 8 has moderate centrality, and density but appears less frequently in the dataset. These clusters offer a structured view of the dataset, facilitating the identification of trends and patterns within distinct health-related domains.

### Most Frequent Word

**FIGURE 5:**
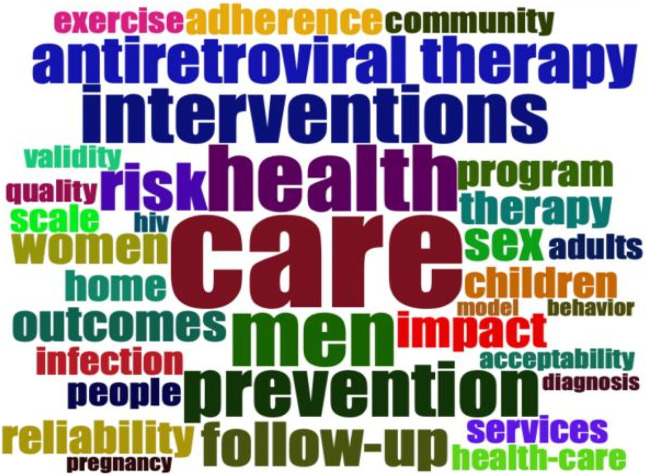
Word cloud of authors keywords use in digital intervention, HIV self-testing and linkage to care researches (Tanzania, 2023).

Keyword analysis contributes to the identification of topics in a particular field. This section performs co-occurrence analysis, burst detection of most frequent words and identification of research hotspots, Frontier and trends. Regarding the most frequent words Figure 5 presents the word cloud related to digital intervention, HIV self-testing and linkage to care. The figures highlight the most frequent keywords used by authors in their publications, the word with the highest number of occurrences being Care (Occurrences 40), followed by Health (Occurrences 24) and Men (Occurrence 23). This illustrates the importance of care in the field of HIV testing. It is surprising to notice that “follow up” is only ranked 8th with 16 occurrences (see Supplementary File S2). This shows how linkage to care and follow up services is problematic in the HIV prevention field.

### Content Analysis

Having laid the groundwork with a thorough bibliometric analysis exploring the landscape of digital interventions in HIV self-testing and linkage to care, this study delves deeper through a subsequent content analysis. In content analysis, we navigate through distinct themes that emerge from the literature, shedding light on the multifaceted landscape of digital intervention. Our exploration begins with an examination of various digital interventions encompassing mHealth, eHealth, mobile health, text messaging and online platforms. We then shift our focus to the nuanced dynamics of HIV self-testing, linkage to care and we scrutinize the implementation models, frameworks and evaluation methodologies.

#### Digital Interventions on HIV Self-Testing and Linkage to Care

The study explores various types of digital interventions used in HIV testing and linkage to care, such as mHealth, eHealth, mobile health, text messaging, and online platforms [40–43]. The content analysis reveals that digital interventions have shown promise in improving HIV testing rates and facilitating linkage to care. Some studies have reported increased testing coverage and early diagnosis rates due to the accessibility and convenience offered by digital interventions. Challenges in implementing digital interventions include technological barriers, privacy concerns, and the need for tailored interventions for specific populations. Strategies in implementing successful implementation include user-centered design, integration into existing healthcare systems, and ensuring equity in access to digital interventions. The findings of this study align with previous research that has highlighted the positive impact of digital interventions on HIV testing and linkage to care [44]. Other studies have also emphasized the need for addressing implementation challenges and tailoring interventions to specific populations. However, it is important to note that the effectiveness and acceptance of digital interventions may vary across different settings and populations, as highlighted by some studies.

#### HIV Self-Testing

This theme focuses on the benefits and limitations of HIV self-testing as a tool for increasing testing coverage. The content analysis reveals that HIV self-testing has gained acceptance and has been shown to improve testing rates, particularly among hard-to-reach populations [45, 46]. Studies highlight the importance of providing accurate information, counseling, and support services to individuals using self-testing kits. Concerns related to accuracy, linkage to care, and the potential for missed opportunities for counseling and prevention are also discussed. The findings of this study are consistent with previous research indicating the acceptability and effectiveness of HIV self-testing in increasing testing coverage [46]. Other studies have also highlighted the need for ensuring the accuracy and reliability of self-testing kits and the importance of linkage to care for those testing positive. Discussion around the need for comprehensive support services and counseling align with findings from other studies as well.

#### Linkage to Care

This theme explores the importance of linkage to care for individuals diagnosed with HIV and the barriers and facilitators associated with it. The content analysis reveals that timely and effective linkage to care is crucial for improving health outcomes and reducing transmission. Barriers to linkage to care include stigma, lack of awareness, transportation issues, and healthcare system-related factors [39]. Facilitators include targeted interventions, peer support, and the use of digital platforms for appointment reminders and tracking.

#### Implementation and Evaluation

This theme focuses on models, frameworks, and evaluation methodologies used in implementing and evaluating digital interventions, HIV self-testing, and linkage to care programs. The content analysis reveals the use of various implementation models, such as the RE-AIM framework and the Diffusion of Innovations theory. Evaluation methodologies include quantitative measures (e.g., testing rates, linkage rates) as well as qualitative assessments of user experiences and program impact. Cost-effectiveness and ethical considerations in the implementation and evaluation process are also discussed.

## Discussion

This bibliometric analysis provides a comprehensive picture of the structure and the development of digital health in HIVST and linkage to care. To the best of our knowledge this is the first study to use quantitative approach and cover the most recent published literature. The analysis shows that academic interest in digital health interventions in HIVST has increased considerably with an annual growth rate of nearly 12.25%. Noteworthy is the substantial increase in articles, particularly in 2015 and 2021, indicating periods of heightened research activity. About 289 documents were included in this analysis.

Despite this fast-growing interest in digital health in HIVST, the field is still fragmented. The 289 articles retrieved are split between 2,146 authors and 149 sources. The analysis further reveals the influence and enduring relevance of specific works, particularly systematic reviews [7, 11, 12, 15, 20, 37–39, 44]. The fragmentation is also highlighted by the collaboration analysis, which shows very long-lasting collaborations. Authors collaborate frequently (with an average of 8.17 authors per document). The field appears to be concentrated in the United States as it emerges as the major contributor, engaging in widespread collaborations, notably with Kenya (10 collaborations), South Africa (16 collaborations) and the United Kingdom (17 collaborations). Australia, Canada, South Africa and the United Kingdom also play significant roles in international collaborations. The proliferation of digital health in HIVST research in these countries could be justified by the fact that they were early adopters of digital technologies in HIV prevention and treatment [37–39, 47].

The thematic map developed through co-word analysis helps to identify the most consolidated themes and provide evidence on the emerging ones. The analysis offers insights into trends and patterns within distinct health-related domains. The niche topic in this analysis was found to be “User Acceptance.” The key words and terms associated with user acceptance of health technologies and predictive models form a distinct and focused theme within the larger research field. While this cluster may not appear as frequently as some other themes, it is densely interconnected, suggesting that there is a concentrated body of research on this specific topic, even if it is not as widespread as other themes.

This work provides essential insights for practitioners, scholars, decision-makers, and policymakers involved in the digital transformation, HIV self-testing and linkage to care fields. The study also contributed in the academic, the study results showed that digital intervention, HIV self-testing and linkage to care keeps as a hot topic, and the needs of more works from emerging economies like Africa countries is fundamental to an in-depth understanding of the particularities of each country. However, it is crucial to acknowledge the study’s limitations. The choice for a database, in this case, WoS, could limit the search and poses a potential gap in coverage. Also, the use of keywords for the literature search introduces the possibility of overlooking relevant papers that may not align with the chosen terminology. In addition, while the provided information offers valuable insights into scientific production and trends, it falls short of facilitating a comprehensive assessment of publication quality. Acknowledging this limitation will be essential for refining methodologies and advancing a more comprehensive understanding of the dynamic field of digital health in HIVST and linkage to care.

### Conclusion

The bibliometric method adopted in this study was very useful for investigating and providing comprehensive picture of digital health interventions in HIVST and linkage to care, especially due to various techniques used performance analysis, collaboration analysis and science mapping. Although a bibliometric method is objective and re-producible, it also implies a less detailed understanding than qualitative techniques. For example, the citation and co-citation analyses show the most cited references, but do not reveal the reason for the citation. Again, the conceptual map highlights the main themes, but does not allow for an in-depth analysis of the contents of each paper. The key insights presented included the following [1]: the most productive authors, institutions, and countries in digital health intervention and HIV self-testing [2] the most cited authors, countries and journals outlets in the area.

## Abbreviations

HIV, Human Immunodeficiency Virus; HIVST, Human Immune Virus Self Testing; WoS, Web of Science; WHO, World Health Organization; MSM, Men who have sex with men; UNAIDS, The Joint United Nations Programme on HIV/AIDS; AIDS, Acquired Immune Deficiency Syndrome; UN, United Nations; STI, Sexual Transmitted Infections.
