# Corrigendum: Digital Intervention Services to Promote HIV Self-Testing and Linkage to Care Services: A Bibliometric and Content Analysis—Global Trends and Future Directions

**DOI:** 10.3389/phrs.2024.1607471

**Published:** 2024-06-03

**Authors:** Frank Mhando, Marwa Nyankomo, Christa Hall, Kelia Olughu, Mbuzeleni Hlongwa, Samuel Janson, Love O. Idahosa, Genae Hatcher, Donaldson F. Conserve

**Affiliations:** ^1^ Johannesburg Business School, University of Johannesburg, Johannesburg, South Africa; ^2^ Ifakara Health Institute, Bagamoyo Research and Training Centre, Bagamoyo, Tanzania; ^3^ Milken Institute School of Public Health, The George Washington University, Washington, DC, United States; ^4^ School of Nursing and Public Health, University of KwaZulu-Natal, Durban, South Africa; ^5^ Public Health, Societies and Belonging, Human Sciences Research Council, Pretoria, South Africa; ^6^ Department of Economics, The University of Warwick, Coventry, United Kingdom

**Keywords:** digital health, mobile health, HIV self-testing, bibliometric analysis, linkage to care

Genae Hatcher was not included as an author in the published article. The corrected **Author Contributions** Statement appears below.

